# Cardiac hypertrophy with obesity is augmented after pregnancy in C57BL/6 mice

**DOI:** 10.1186/s13293-019-0269-z

**Published:** 2019-12-16

**Authors:** Chen Che, Kayla Dudick, Robin Shoemaker

**Affiliations:** University of Kentucky, Department of Dietetics and Human Nutrition, 203 Funkhouser Bldg, Lexington, KY 40506-0054 USA

**Keywords:** Pregnancy, Maternal, CVD, Remodeling, Obesity, Cardiac hypertrophy

## Abstract

**Background:**

Over a third of reproductive-age women in the USA are obese, and the prevalence of cardiovascular disease (CVD) is rising in premenopausal women. Cardiac hypertrophy is an independent predictor of CVD. In contrast to pregnancy, where transiently increased left ventricular (LV) mass is not associated with cardiac damage, obesity-mediated cardiac hypertrophy is pathological. There is a paucity of data describing the effect of obesity during pregnancy on maternal cardiovascular health. The purpose of this study was to determine the long-term effect of obesity during pregnancy on cardiac function and structure in mice.

**Methods:**

Female C57BL/6 J mice were fed a high-fat (HF) or a low-fat (LF) diet for 20 weeks. After 4 weeks, LF- and HF-fed female mice were either crossed with males to become pregnant or remained non-pregnant controls. Following delivery, pups were euthanized, and females maintained on respective diets. After 20 weeks of diet feeding, cardiac function was quantified by echocardiography, and plasma leptin and adiponectin concentrations quantified in LF- and HF-fed postpartum and nulliparous females. mRNA abundance of genes regulating cardiac hypertrophy and remodeling was quantified from left ventricles using the NanoString nCounter Analysis System. Cardiac fibrosis was assessed from picrosirius red staining of left ventricles.

**Results:**

HF-fed postpartum mice had markedly greater weight gain and fat mass expansion with obesity, associated with significantly increased LV mass, cardiac output, and stroke volume compared with HF-fed nulliparous mice. Plasma leptin, but not adiponectin, concentrations were correlated with LV mass in HF-fed females. HF feeding increased LV posterior wall thickness; however, LV chamber diameter was only increased in HF-fed postpartum females. Despite the marked increase in LV mass in HF-fed postpartum mice, mRNA abundance of genes regulating fibrosis and interstitial collagen content was similar between HF-fed nulliparous and postpartum mice. In contrast, only HF-fed postpartum mice exhibited altered expression of genes regulating the extracellular matrix.

**Conclusions:**

These results suggest that the combined effects of pregnancy and obesity augment cardiac hypertrophy and promote remodeling. The rising prevalence of CVD in premenopausal women may be attributed to an increased prevalence of women entering pregnancy with an overweight or obese BMI.

## Background

Cardiovascular disease (CVD), the number one cause of death in both women and men in the USA [1], is manifested differently in women compared with men. Differences in the types of CVD, timing of onset, and mortality rate have been largely attributed to sex hormones, and more recently, sex chromosomes [2]. However, discrepancies in effects of hormones to protect against CVD in randomized-controlled trials [3] indicate that other sex-specific factors also contribute to differential cardiovascular function between males and females. Aside from sex hormones and sex chromosomes, the physiologic experience of pregnancy is unique to females. Pregnancy requires a profound, but transient, adaptation of the cardiovascular system, including dramatic increases in blood volume, cardiac output (CO), and left ventricular (LV) mass [4]. Complications during pregnancy impacting the cardiovascular system, such as gestational hypertension or peripartum cardiomyopathy, are associated with increased risk of developing CVD later in life [5].

Obesity is strongly associated with pregnancy complications [6]. The prevalence of obesity in the USA is increasing in women of reproductive age, with approximately 36.5% of women aged 20–39 having a body mass index (BMI) of greater than 30 [7]. Obesity augments traditional cardiovascular risk factors, such as hypertension, and is directly associated with cardiac hypertrophy [8]. Cardiac hypertrophy, a compensatory enlargement of the ventricles as a result of sustained pressure or volume overload, is an independent predictor of CVD [9]. With pregnancy, cardiac hypertrophy occurs due to increased hemodynamics as well as hormonal changes [10–12]. Pregnancy-induced cardiac hypertrophy is assumed to be transient and is not associated with cardiac damage [4, 13]. In contrast, obesity-mediated cardiac hypertrophy is pathological [14], and not usually reversible [15].

Despite the well-known associations between obesity, pregnancy complications, and subsequent maternal CVD, there is a paucity of data describing the effects of obesity on cardiovascular function during and after pregnancy. In humans [16] and experimental animals [17], obesity during pregnancy increases blood pressure. The few studies where cardiovascular function was assessed during pregnancy in obese women report increased LV mass [16] and impaired contractile function [18, 19]. No studies have assessed the longitudinal effects of obesity during pregnancy on cardiovascular function. Specifically, whether the combined hypertrophic effects of obesity and pregnancy adversely impact cardiac structure and function after pregnancy are not known. The purpose of the current study was to define the effects of obesity during pregnancy on postpartum cardiac function and structure in a mouse model of high-fat feeding.

## Methods

### Experimental animals

All studies using mice were approved by the Institutional Animal Care and Use Committee (IACUC) at the University of Kentucky and were conducted in accordance with the National Institutes of Health (NIH) Guide for the Care and Use of Laboratory Animals. Female C57BL/6 J mice (8 weeks of age; Jackson Laboratory, Bar Harbor, ME, stock # 000664) were randomly assigned to receive, ad libitum, either a high-fat (HF; 60% kcal from fat; D12492, Research Diets, New Brunswick, NJ) or a control low-fat (LF, 10% kcal from fat; D12450B, Research Diets Inc.) diet for 20 weeks (*n* = 20 mice/diet group). The control LF diet was purified and ingredient-matched to the HF diet, and the fat source for both diets was soybean oil and lard (where lard comprises the excess fat in the HF diet). The energy densities of the LF and HF diet are 3.82 and 5.21 kcal/g, respectively (see Additional file 1: Table S1 for macronutrient composition of the diets). Body weight was quantified weekly throughout the study using an Ohaus portable digital scale. At 4 weeks of diet feeding, female mice were randomly assigned to either become pregnant or remain non-pregnant controls (LF, *n* = 10 mice/group; HF, *n* = 9 mice in the non-pregnant group and *n* = 11 mice in the pregnant group). In the pregnancy group, female mice were crossed with male mice of the same strain and diet. Visibly pregnant females were separated from males, maintained on LF or HF diet in single-housing until after delivery (where pups were euthanized to control for lactation as an additional variable), and returned to group housing (4–5 mice of the same sex and diet group) for the duration of the study. Females who became pregnant delivered pups at a mean of 9.2 weeks on diet. Non-pregnant control mice were group-housed for the duration of the study. At week 20 of diet feeding, fat and lean mass were quantified by EchoMRI (Echo Medical Systems, Houston, TX), and cardiac function and structure were quantified by echocardiography in LF- and HF-fed postpartum or non-pregnant (nulliparous) female mice. At the study endpoint, mice were anesthetized with ketamine/xylazine (100/10 mg/kg, i.p.) for exsanguination and tissue harvest. Tissues were snap frozen in liquid nitrogen and stored at − 80 °C until analysis (see Additional file 1: Figure S1 for experimental design).

### Echocardiography

Echocardiography was performed on isoflurane-anesthetized LF- and HF-fed female mice at week 20 of diet feeding in postpartum (mean of 10.5 weeks following delivery) and nulliparous controls. Mice were anesthetized using 2–4% isoflurane (at effect) according to their size and then transferred to a heated platform (37 °C) with 1–2% isoflurane supplied via a nose cone. The hair on the chest region was shaved and removed, and electrode cream was applied on the front and hind limbs before being secured with electrical tape to electrodes on the platform. Respiration rate (RR) and heart rate (HR) were monitored and adjusted to a certain range across all mice by titrating isoflurane levels. An RR of 100 times/min and HR of 400 beats/min were targeted. Images of the cross-sectional view of the left ventricle (LV) at the papillary muscle-level in parasternal short-axis (PSAX) view were obtained in M-mode using an M550 transducer under the cardiology package on a Vevo 3100. Images were analyzed using VevoLab software using LV trace methodology. The following parameters were measured over three cardiac cycles: thickness of the interventricular septum (IVS), LV interior diameter (LVID), and LV posterior wall (LVPW) and used to make the following calculations (via the VevoLab software): ejection fraction (EF; 100 × ((LV Vol;d – LV Vol; s)/LV Vol;d)), fractional shortening (FS; 100 × ((LVID; d – LVID;s)/LVID;d)), stroke volume (SV; LV Vol;d – LV Vol;s), LV mass (1.053 × (LVID;d + LVPW;d + IVS;d)^3^ – LVID;d^3^), and cardiac output (CO; SV × HR).

### Tissue RNA extraction and gene expression analysis

Approximately 20 mg of the left ventricle was used to extract total RNA using the Maxwell RSC (Promega, Madison, WI). RNA concentrations and purity were determined using an Agilent 2100 Bioanalyzer (Agilent, Santa Clara, CA). All samples had an RNA integrity number (RIN) > 8.5 (average = 9.35). mRNA abundance was measured with the NanoString nCounter Analysis System (NanoString Technologies, Seattle, WA) using a custom nCounter CodeSet of 39 genes selected by the investigators involved in cardiac hypertrophy, fibrosis, and angiogenesis, and four reference genes (glyceraldehyde-3-phosphate dehydrogenase [*Gapdh*], eukaryotic translation elongation factor 1 epsilon 1 [*Eef1e1*], ribosomal protein L4 [*Rpl4*], and tyrosine 3-monooxygenase/tryptophan 5-monooxygenase activation protein zeta [*Ywhaz*]). The NanoString nCounter gene expression system is a multiplexed assay that uses a combination of unique capture probes and color-coded reporter probes to capture and count individual mRNA transcripts with high sensitivity and tight correlation to real-time PCR [20, 21]. Fifty nanograms of RNA of each sample was hybridized to the target-specific capture and reporter probes in the CodeSet according to the manufacturer’s instructions. Samples were cooled to 4 °C, loaded into nCounter SPRINT cartridges and then analyzed using the nCounter Gene Expression Assay. Raw data were normalized by creating scaling factors for the sum of the positive controls and the geometric mean of the four housekeeping genes [20, 21]. Data represent the mean of normalized counts. A complete list of genes in the custom CodeSet is included in Additional file 1: Table S2. One gene was excluded from analysis, *Agtr2*, because mRNA counts were below that of positive controls.

### Quantification of interstitial collagen content

Hearts were fixed in 10% formalin overnight, dehydrated in grades of ethanol, and paraffin embedded. Starting at the papillary muscle, cross sections (5 μm) were prepared every 50 μm. Sections were deparaffinized, rehydrated, and incubated with Picrosirius solution for 1 h. Staining was followed by washing with acidic water, dehydration, and mounting. Images were acquired from 3 sections per mouse under bright-filed microscopy at × 20 using a Nikon Eclipse 80i microscope. Interstitial collagen content was quantified by ImageJ software (NIH) using color thresholding in 5 random fields distributed across the LV wall per section. Interstitial collagen content is presented as the percentage of pixels with red staining of the total number of pixels per image. Data are reported as the mean percentage of collagen staining per mouse (*n* = 3 mice per group).

### Plasma parameters

Plasma adiponectin concentrations were quantified by ELISA using a commercially available kit (catalog number MRP300, R&D systems, Minneapolis, MN). Plasma leptin concentrations were quantified by ELISA using a commercially available kit (catalog number 22-LEPMS-E01, ALPCO, Salem, NH).

### Statistical analyses

Data are presented as mean ± SEM. Statistical analyses were performed using SigmaPlot version 12.3. All data passed normality or equal variance tests or logarithmic transformation was used to achieve normality. Two-tailed Student’s *t* tests were used for the analysis of data between two groups. For 2-factor analysis, a two-way ANOVA was used to analyze endpoint measurements with between-group factors of pregnancy and diet, followed by Holm-Sidak for post hoc pairwise analyses. Correlation analyses were performed between plasma parameters and LV mass. Values of *P* < 0.05 were considered to be statistically significant.

## Results

### Weight gain from HF-diet feeding is exaggerated in female mice after pregnancy

**Fig. 1:**
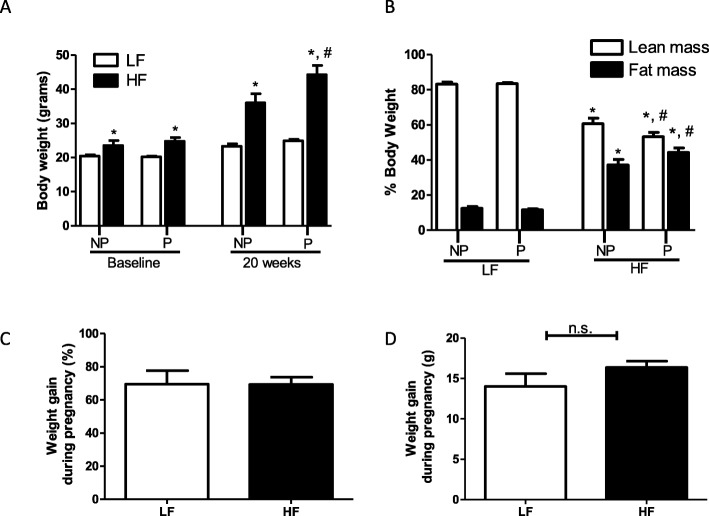
Weight gain with high-fat feeding is exacerbated after pregnancy. **a** Body weight of low-fat (LF)– and high-fat (HF)–fed mice at baseline (4 weeks of diet feeding, prior to pregnancy) and after 20 weeks of diet feeding in nulliparous (NP) and postpartum (P) female mice (mean of 10.5 weeks after delivery). **b** Lean and fat mass (as percentage of body weight) of LF- and HF-fed mice after 20 weeks of diet feeding in NP and P female mice (mean of 10.5 weeks after delivery). **c** Percent weight gain and (**d**) grams gained during pregnancy in LF- and HF-fed mice. Data are mean + SEM from *n* = 9–11 mice per group. *P < 0.01 compared with LF within group using 2-way ANOVA followed by Holm-Sidak pairwise analysis; ^#^*P* < 0.05 compared with NP within diet group using 2-way ANOVA followed by Holm-Sidak pairwise analysis

At baseline (4 weeks of diet feeding, prior to pregnancy), HF-fed mice had increased body weight compared with LF-fed mice (*P* < 0.001), and there was no difference within diet group in the baseline body weight (Fig. 1a). Weight gain with pregnancy (as both percentages of body weight gained and in grams) was similar in LF-fed and HF-fed mice (Fig. 1c, d). After 20 weeks of diet feeding, body weight was significantly increased in HF-fed compared with LF-fed female mice (Fig. 1a, *P* < 0.001). HF-fed females had greater fat mass and less lean mass (as a percentage of body weight) compared with LF controls (Fig. 1b, *P* < 0.001). In LF-fed mice that became pregnant after 1 month of diet feeding, there was no difference in body weight, or lean/fat mass percentage of body weight at 20 weeks of diet feeding compared with nulliparous LF-fed mice. In contrast, HF-fed mice that became pregnant after 1 month of diet feeding had increased body weight (Fig. 1a, *P* < 0.01), decreased lean mass, and increased fat mass (as a percentage of body weight, *P* < 0.05) after 20 weeks of diet feeding compared with nulliparous HF-fed mice (Fig. 1b).

### Cardiac output (CO) and stroke volume (SV) increase with HF feeding in postpartum, but not nulliparous female mice

**Fig. 2:**
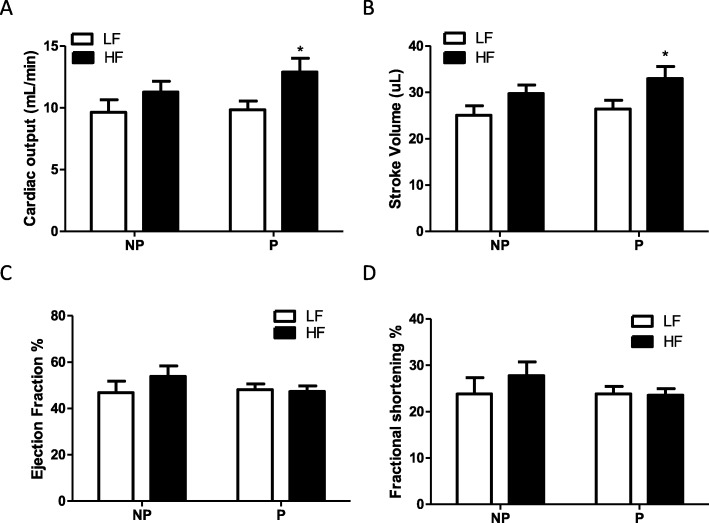
High-fat-fed postpartum mice have increased cardiac output and stroke volume compared with high-fat-fed nulliparous mice. **a** Cardiac output. **b** Stroke volume. **c** Ejection fraction. **d** Fractional shortening after 20 weeks of low-fat (LF) or high-fat (HF) feeding in female nulliparous (NP) or postpartum (P) mice. Data are mean + SEM from *n* = 9–11 mice per group. *P < 0.05 compared with LF within group using 2-way ANOVA followed by Holm-Sidak pairwise analysis

Cardiac function was quantified by echocardiography after 20 weeks of diet feeding in postpartum and nulliparous female mice. There was an overall effect of HF feeding to increase CO and SV (*P* < 0.05); however, pairwise statistical analysis revealed that this effect was significant only in HF-fed postpartum (*P* < 0.05), and not HF-fed nulliparous mice (*P* > 0.05) compared with respective LF controls (Fig. 2a, b). In contrast, there was no effect of HF feeding on ejection fraction (EF) or fractional shortening (FS) in postpartum or nulliparous female mice (Fig. 2c, d). In LF-fed mice, there was no difference in any functional parameter in postpartum compared with nulliparous mice (Fig. 2a, b, c, d).

### Cardiac structure is augmented in HF-fed postpartum vs nulliparous mice

**Fig. 3:**
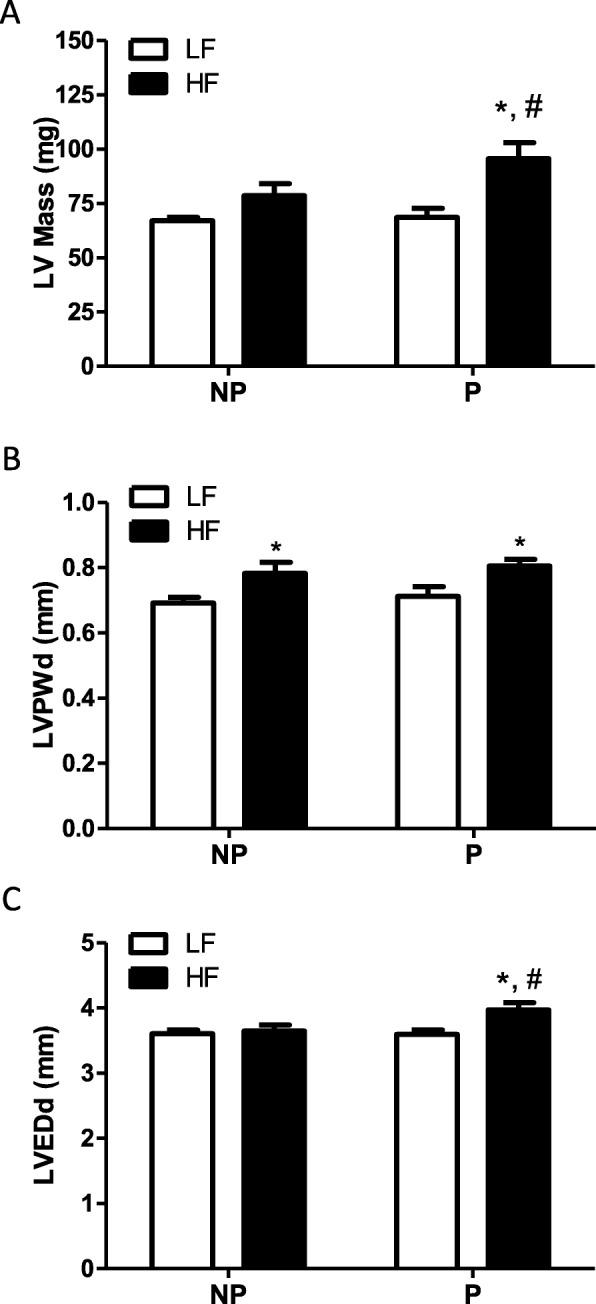
High-fat-fed postpartum mice exhibit increased left ventricular mass with enlargement of the left ventricular chamber. **a** Left ventricular (LV) mass. **b** LV posterior wall diameter (LVPWd). **c** LV end-diastolic diameter (LVEDd) in low-fat (LF)– and high-fat (HF)–fed nulliparous (NP) and postpartum (P) mice after 20 weeks of diet feeding. HF feeding increases LVPWd in the absence of LV chamber enlargement in NP mice. HF-fed postpartum mice have significantly increased LV mass accompanied by LV chamber dilation. Data are mean + SEM from *n* = 9–11 mice per group. *P < 0.01 compared with LF within group using 2-way ANOVA followed by Holm-Sidak pairwise analysis; ^#^*P* < 0.05 compared with NP within diet group using 2-way ANOVA followed by Holm-Sidak pairwise analysis

In HF-fed nulliparous mice, there was a trend to increase LV mass compared with LF controls, but the effect was not statistically significant using pairwise analysis (Fig. 3a; *P* = 0.151). In contrast, LV mass was markedly increased in postpartum mice fed a HF diet compared with LF-fed postpartum controls (Fig. 3a; *P* < 0.001). Moreover, LV mass in HF-fed postpartum mice was significantly increased compared with LV mass of HF-fed nulliparous mice (Fig. 3a, *P* < 0.05). In both postpartum and nulliparous mice, HF feeding increased the LV posterior wall diameter (LVPWd) compared with LF-fed controls (Fig. 3b; *P* < 0.001). However, LV chamber size, assessed as LV end-diastolic diameter (LVEDd) was increased with HF feeding only in postpartum mice (*P* < 0.01), and LVEDd was significantly greater in HF-fed postpartum compared with HF-fed nulliparous mice (Fig. 3c; *P* < 0.05). In LF-fed mice, there were no differences in LV mass, posterior wall thickness, or LV diameter in postpartum vs nulliparous mice (Fig. 3a, b, c). HF feeding increased absolute heart weight in postpartum, but not nulliparous, mice compared with LF-fed counterparts (*P* < 0.05; LF nulliparous, 0.112 + 0.004; LF postpartum, 0.116 + 0.005; HF nulliparous, 0.116 + 0.003; HF postpartum, 0.14 + 0.008 g). HF-fed mice had decreased heart to body weight ratio compared with LF-fed mice (*P* < 0.05), with no effect of nulliparity compared with postpartum (LF nulliparous, 0.49 + 0.01; LF postpartum, 0.46 + 0.01; HF nulliparous, 0.33 + 0.02; HF postpartum, 0.33 + 0.01% heart to body weight).

We quantified plasma concentrations of leptin and adiponectin, as these adipocyte-mediated hormones have been demonstrated in vitro and in vivo to have direct effects on cardiac hypertrophy [14, 22]. Plasma leptin concentrations were markedly increased with HF feeding (*P* < 0.001, Additional file 1: Table S3), although there was no difference between nulliparous and postpartum mice. Plasma leptin concentrations in HF-fed mice were positively correlated with LV mass in both nulliparous (*r* = 0.88, *r*^*2*^ = 0.78, *P* < 0.01) and postpartum mice (*r* = 0.77, *r*^*2*^ = 0.59, *P* < 0.01). In contrast, there was no effect of the HF diet on plasma adiponectin concentrations (Additional file 1: Table S3). Although there was a modest effect of pregnancy to increase plasma adiponectin, pairwise analysis revealed that this increase was significant in LF-fed but not HF-fed mice (*P* < 0.05, Additional file 1: Table S3). Plasma adiponectin concentrations were not correlated with LV mass in any of the groups.

### Expression profile of genes regulating cardiac remodeling and hypertrophy is altered in HF-fed postpartum mice

To determine the gene profile associated with changes in the cardiac structure of HF-fed postpartum mice, we quantified mRNA abundance of genes regulating fibrosis, extracellular matrix (ECM) remodeling, cardiac hypertrophy, angiogenesis, estrogen receptors, and the renin-angiotensin system using NanoString nCounter gene expression analysis in the left ventricles of nulliparous and postpartum mice after 20 weeks of diet feeding. A complete list of the genes included in the CodeSet is included in Additional file 1: Table S2.

#### Fibrosis

**Fig. 4:**
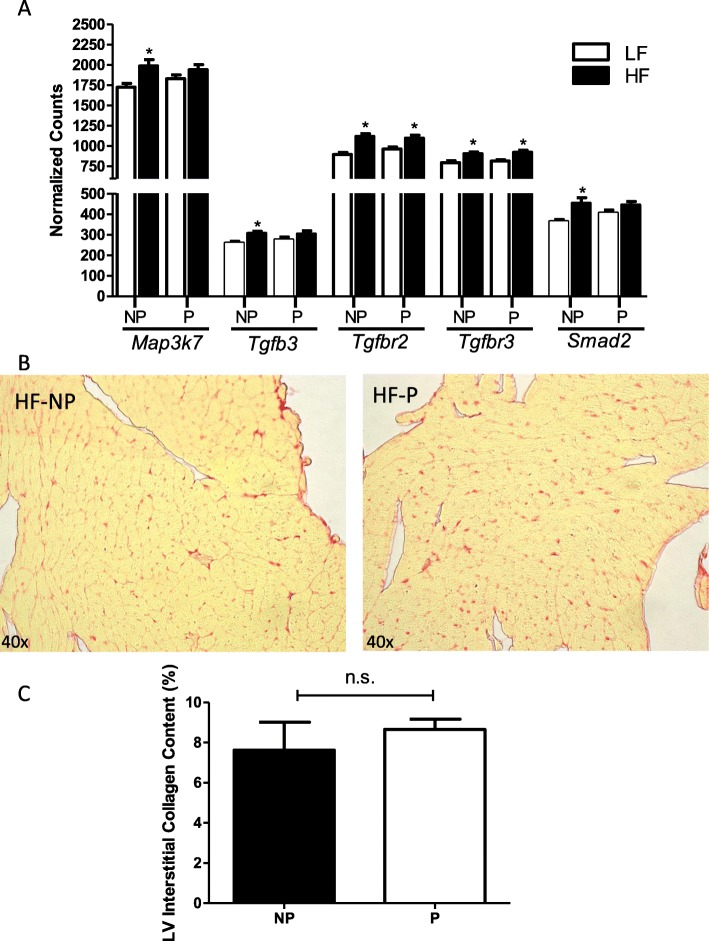
Cardiac fibrosis with high-fat feeding is not augmented in postpartum mice. **a** mRNA abundance of fibrosis-related genes (*Map 3k7*, *Tgfb3*, *Tgfbr2*, *Tgfbr3*, and *Smad2*) in nulliparous (NP) and postpartum (P) mice fed a low-fat (LF) and high-fat (HF) diet for 20 weeks*.* mRNA abundance of genes left ventricles was quantified using a custom CodeSet from NanoString and analyzed on an nCounter Analysis System. Data are expressed as counts of mRNA transcripts, normalized to the geometric mean of counts of four housekeeping genes (*Gapdh*, *Eef1e1*, *Rpl4*, and *Ywhaz*). A complete list of genes included in the custom CodeSet is available in Additional file 1: Table S2. Data are mean + SEM from *n* = 9 (LF, NP, and P), *n* = 7 (HF, NP), and *n* = 11 (HF, P). **b** Representative images (× 40) of picrosirius red–stained sections and **c** quantification of interstitial collagen content in the LV wall of NP- and P HF-fed mice. Data are mean + SE from the average of 5 fields per section (3 sections per mouse, and *n* = 3 mice per group). *P < 0.05 compared with LF using 2-way ANOVA followed by Holm-Sidak pairwise analysis

There was an overall effect of HF feeding to increase cardiac mRNA abundance of the fibrosis-related genes mitogen-activated protein kinase kinase kinase 7 (*Map 3k7*; *P* < 0.01), transforming growth factor beta 3 (*Tgfb3*; *P* < 0.01), transforming growth factor beta receptor 2 (*Tgfbr2*; *P* < 0.001), transforming growth factor beta receptor 3 (*Tgfbr3*; *P* < 0.001) and *Smad2* (*P* < 0.001) in both nulliparous and postpartum female mice compared with LF controls (Fig. 4a). However, pairwise statistical analysis revealed that only *Tgfbr2* and *Tgfbr3* were significantly increased with HF feeding in both nulliparous and postpartum mice. Effects of HF feeding to increase mRNA abundance of *Map 3k7*, *Tgfb3*, and *Smad2* were only statistically significant in HF-fed nulliparous, and not HF postpartum mice compared with LF controls using pairwise comparisons (*P* > 0.05). There was no effect of pregnancy on mRNA abundance of genes regulating fibrosis in either LF- or HF-fed mice. We quantified interstitial collagen content in picrosirius red–stained left ventricles of HF-fed nulliparous and postpartum mice, and there was no difference between groups in collagen staining (Fig. 4b, c; *P* = 0.5).

**Fig. 5:**
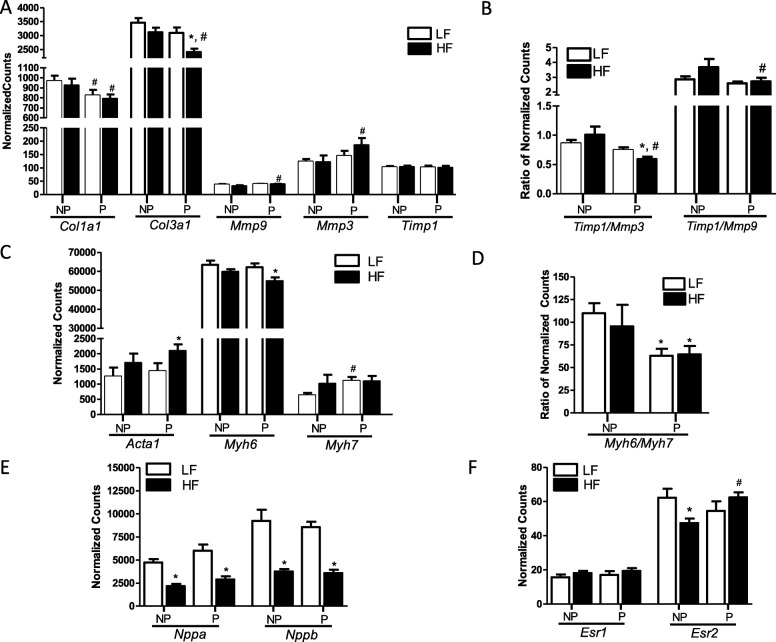
mRNA abundance of key genes regulating extracellular matrix and cardiac hypertrophy in left ventricles of low fat- and high-fat-fed nulliparous and postpartum mice. **a** mRNA abundance of genes regulating the extracellular matrix (ECM): *Col1a1*, *Col3a1*, *Mmp9*, *Mmp3*, and *Timp1.* **b** Ratios of *Timp1*to *Mmp3* and *Mmp9.* **c** mRNA abundance of fetal gene program genes, *Acta1*, *Myh6*, and *Myh7.* **d** Ratio of *Myh6* to *Myh7*, a marker of fetal gene reactivation. **e** mRNA abundance of natriuretic peptides A and B (*Nppa and Nppb*). **f** mRNA abundance of estrogen receptors α and β (*Esr1 and Esr2*). mRNA abundance of genes from left ventricles of low-fat (LF)– and high-fat (HF)–fed nulliparous (NP) and postpartum (P) mice was quantified using a custom CodeSet from NanoString and analyzed on an nCounter Analysis System. Data are expressed as counts of mRNA transcripts, normalized to the geometric mean of counts of four housekeeping genes (*Gapdh*, *Eef1e1*, *Rpl4*, and *Ywhaz*). A complete list of genes included in the custom CodeSet is available in Additional file 1: Table S2. Data are mean + SEM from *n* = 9 (LF, NP, and P), *n* = 7 (HF, NP), and *n* = 11 (HF, P). *P < 0.05 compared with LF using 2-way ANOVA followed by Holm-Sidak pairwise analysis. ^#^*P* < 0.05 compared with NP using 2-way ANOVA followed by Holm-Sidak pairwise analysis

In contrast, there was an effect of pregnancy to alter genes involved in the remodeling of the ECM. There was an overall effect of pregnancy to increase mRNA abundance of matrix metallopeptidases 3 and 9 (*Mmp3 Mmp9; P < 0.05*) compared with nulliparous mice. Pairwise analysis revealed that postpartum mice fed with HF but not LF diet had increased mRNA abundance of *Mmp3* and *Mmp9* compared with nulliparous controls (Fig. 5a; *P* < 0.05). mRNA abundance of metallopeptidase inhibitor 1 (*Timp1*) was similar between groups (Fig. 5a), but the ratio of *Timp1* to *Mmp3* and to *Mmp9* was reduced in HF-fed postpartum mice compared with HF-fed nulliparous mice (Fig. 5b; *P* < 0.05). This was accompanied by reduced mRNA abundance of collagen type I alpha 1 (*Col1a1*) in LF- and HF-fed postpartum compared with nulliparous mice (Fig. 5a; *P* < 0.05). Further, there was an overall effect of pregnancy to reduce mRNA abundance of collagen type III alpha 1 (*Col3a1; P* < 0.01), but pairwise analysis demonstrated a significant reduction in *Col3a1* mRNA abundance with HF feeding only in postpartum and not nulliparous mice (Fig. 5a; *P* < 0.01).

#### Fetal gene program

Induction of genes predominately expressed during fetal cardiac development, termed the fetal gene program (FGP), occurs with pathological cardiac hypertrophy and impaired cardiac function [23]. Therefore, we quantified mRNA abundance of alpha 1 actin (*Acta1*) beta actin (*Actb*), myosin heavy chain 6 (*Myh6*) myosin heavy chain 7 (*Myh7*) sarco/endoplasmic reticulum Ca^2+^ −ATPase (*SERCA2*) and phospholamban (*Pln*) to assess the induction of the PGP in hearts from LF- and HF-fed postpartum and nulliparous mice. There was an overall effect of HF feeding to increase mRNA abundance of *Acta1* (*P* < 0.05); this effect was only significant in HF-fed postpartum, not nulliparous, mice after pairwise analysis (Fig. 5c; *P* < 0.05). Likewise, mRNA abundance of *Myh6* was reduced with HF feeding (*P* < 0.01), with the effect significant in HF postpartum, but not HF-fed nulliparous mice compared with LF mice (Fig. 5c; *P* < 0.01). A decrease in the ratio of *Myh6* to *Myh7* is a marker of fetal gene activation in rodent hearts [24]. mRNA abundance of *Myh7* was moderately increased only in LF-fed postpartum mice compared with LF-fed nulliparous mice (Fig. 5c; *P* < 0.05). However, both LF- and HF-fed postpartum mice exhibited a significant decrease in the *Myh6* to *Myh7* ratio (a reduction of 42% and 32%, respectively; Fig. 5d; *P* < 0.001) compared with nulliparous controls. There was no effect of diet or pregnancy to alter the expression of *Actb*, *SERCA2*, or *Pln* (Additional file 1: Table S2).

#### Natriuretic peptides

Natriuretic peptides are reported to have anti-hypertrophic and anti-fibrotic effects on cardiac tissue [25]. We quantified cardiac mRNA abundance of natriuretic peptides A, B, and C (*Nppa*, *Nppb*, and *Nppc* respectively), and natriuretic peptide receptor 1 (*Npr1*). Both nulliparous and postpartum female mice fed with a HF diet had decreased expression of *Nppa* and *Nppb* compared with LF controls, with no further effect of pregnancy (Fig. 5e; *P* < 0.001). There was no effect of diet or pregnancy to alter gene expression of *Nppc* or *Npr1* (Additional file 1: Table S2).

#### RAS

We quantified the mRNA abundance of components of the RAS, as increased activation of the RAS is strongly associated with cardiac hypertrophy and fibrosis [26]. There was an overall effect of HF feeding to increase mRNA abundance of angiotensin-converting enzyme (*Ace*; Additional file 1: Table S2; *P* < 0.05); however, no significant comparisons were reported with pairwise analysis (*P* > 0.05). There was a trend to increase the expression of angiotensinogen (*Agt*) with HF feeding (*P* = 0.053, Additional file 1: Table S2), but this was not significant. Moreover, neither diet nor pregnancy altered mRNA abundance of any other components of the RAS (Additional file 1: Table S2).

#### Angiogenesis

Dysregulation of angiogenesis is associated with impaired cardiac function with pregnancy [27]. Therefore, we measured mRNA abundance of angiopoietin 1 and 2 (*Angpt1* and *Angpt2* respectively), peroxisome proliferative–activated receptor gamma coactivator 1 alpha (*Ppargc1a*) and vascular endothelial growth factor A (*Vegfa*), as these genes are reported to regulate cardiac angiogenesis during pregnancy [12, 27]. HF feeding increased mRNA abundance of *Angpt1* in both postpartum and nulliparous mice compared with LF controls (Additional file 1: Table S2; *P* < 0.01). However, there was no effect of diet or pregnancy on the expression of any other angiogenesis-related genes (Additional file 1: Table S2).

#### Estrogen receptors

17β-estradiol is reported to prevent cardiac hypertrophy [28], and the estrogen receptor β (ERβ), encoded by the *Esr2* gene, has been demonstrated to mediate the inhibition of cardiac fibrosis [29]. Therefore, we quantified mRNA abundance of *Esr1* (encoding the estrogen receptor α) and *Esr2* in left ventricles from LF- and HF-fed postpartum and nulliparous mice*.* The mRNA abundance of *Esr1* was not affected by diet or pregnancy (Fig. 5f). Although there was no independent effect of diet or pregnancy on *Esr2*, the interaction between the two factors was statistically significant (*P* < 0.05). Pairwise analysis indicated that mRNA abundance of *Esr2* was reduced with HF feeding in nulliparous mice (*P* < 0.05), but increased with HF feeding in postpartum mice (Fig. 5f; *P* < 0.05).

## Discussion

Obesity is the most common problem in obstetrics associated with pregnancy complications [6], and women with a history of pregnancy complications are at increased risk for future CVD. Obesity independently promotes cardiac hypertrophy, a predictor of CVD [30]; however, it is not known how obesity during pregnancy (where pregnancy is also a hypertrophic stimulus) impacts subsequent cardiac hypertrophy and remodeling. The present study examined the effects of HF feeding during pregnancy on cardiac function and structure in mice postpartum compared with non-pregnant (nulliparous) controls. The major findings of this study are (1) obesity increases post-gestational weight gain and fat mass expansion, (2) obesity increases CO, SV, and LV mass in postpartum mice, with no impairment in systolic function, (3) obesity is associated with fibrosis and increased wall thickness in both postpartum and nulliparous mice; however, obese postpartum mice also exhibit enlarged LV chamber, and (4) the expression profile of genes in left ventricles of obese postpartum mice reflects active ECM remodeling 10 weeks after delivery. These results demonstrate that HF feeding during and after pregnancy promotes cardiac hypertrophy and augments remodeling compared with nulliparous mice.

Pathological cardiac hypertrophy with obesity is distinctly different from hypertrophy occurring in response to physiological stimuli, such as pregnancy or exercise. Cardiac remodeling characterized by either concentric or eccentric geometry is a key feature of pathological hypertrophy. In humans, cardiac remodeling with obesity results in a predominately concentric geometry, where wall thickness is increased with little to no change in chamber size [8]. In comparison with pathological cardiac remodeling, cardiac hypertrophy with pregnancy results in proportional enlargement in chamber size and wall thickness, with minimally altered cardiac geometry. Further, cardiac hypertrophy of pregnancy is reversible as early as 7–10 days postpartum in rodents [31] and returns near baseline within a year in humans [32]. In the current study, lean postpartum mice exhibited no cardiac hypertrophy or altered geometry compared with lean nulliparous mice. With obesity, nulliparous mice had increased wall thickness in the absence of chamber enlargement, reflecting the concentric geometry attributed to obesity. In contrast, obese postpartum mice exhibited an increase in both wall thickness and chamber size. However, despite a marked increase in LV mass in obese postpartum mice, mean wall thickness was equivalent to that of nulliparous mice. The disproportionate increase in chamber diameter and ventricle wall thickness suggests cardiac remodeling with obesity in postpartum mice is characterized by an eccentric, rather than concentric geometry.

Pathological vs physiological hypertrophy is also distinguished by the activation of signaling pathways that promote increased interstitial fibrosis, and ultimately mechanical stiffness. Activation of the TGF-β/TGF-β receptor/Smad signaling pathway is a primary mediator of cardiac fibrosis, resulting in increased deposition of collagen into the ECM [33]. Consistent with published studies [34, 35], obesity increased cardiac mRNA abundance of several fibrosis-related genes, including *Map 3k7*, *Tgfb3*, *Tgfbr2*, *Tgfbr3*, and *Smad2*. Despite the marked increase in LV mass, mRNA abundance of profibrotic genes was not proportionally increased in obese postpartum female mice, and there was no difference in LV interstitial collagen content in obese postpartum compared with nulliparous mice. Moreover, obesity did not increase cardiac gene expression of the RAS (where the RAS is known to stimulate fibrosis). These data are consistent with other studies demonstrating that fibrosis is not a phenotype of pregnancy remodeling [31, 36, 37] and that pregnancy even protects against AngII-induced fibrosis in rats [38].

In humans, sex differences are reported in pathologic cardiac remodeling. In response to pressure overload, women develop less fibrosis with cardiac hypertrophy compared with men [39], and similar findings are reported in experimental animal models of pressure overload [40]. Sex hormones contribute to sex differences in fibrosis, where estrogen attenuates and testosterone promotes cardiac fibrosis [41]. In female mice, ERβ agonism blocked AngII-mediated activation of *Tgfb*, *Mmp2*, and subsequent collagen production [29]. Similarly, physiological hypertrophy of pregnancy is associated with the upregulation of MMPs [36], remodeling enzymes that degrade collagen. Interestingly, we report that mRNA abundance of cardiac *Esr2* was decreased with HF feeding in nulliparous mice but increased in HF-fed postpartum mice. Moreover, this was associated with increased mRNA abundance of *Mmp*s, and decreased *Col1a1* and *Col3a1* mRNA abundance in obese postpartum mice. These changes in gene expression did not translate into reduced interstitial collagen content in HF-fed postpartum compared with nulliparous mice. However, they may explain why the level of fibrosis was similar between the two groups despite the significantly larger LV mass in HF-fed postpartum mice. Studies in rodents have demonstrated that the postpartum period is a time of active ECM remodeling [42]. However, cardiac hypertrophy and changes in ECM gene expression in rodents fed a standard diet revert to pre-pregnancy levels within 7–10 days of delivery [31, 43]. Results from the current study demonstrating differences in ECM gene expression in HF-fed postpartum mice suggest active cardiac remodeling in obese mice 10 weeks after delivery. Whether this is a protective effect against increased fibrosis or deleterious effect on remodeling is not entirely clear. Indeed, non-injurious physiological hypertrophy, as with exercise, is associated with cardiac remodeling and hypertrophy [44]. In a recent study, HF-fed male rats subjected to aortic valve regurgitation (a model of eccentric remodeling) had increased cardiac hypertrophy and decreased survival compared with HF-fed rats without aortic value regurgitation [45], suggesting additive adverse effects of HF feeding and cardiac remodeling. In the current study, given the evidence of cardiac fibrosis in HF-fed postpartum mice, we speculate changes in cardiac structure in HF-fed postpartum mice are not likely to be a positive adaptation. Rather, taken with the ECM gene expression profile, increased LV mass and chamber dilation may reflect the degradation of the ECM.

Induction of the FGP is a marker of pathological cardiac hypertrophy and dysfunction. We observed only mild alterations in the expression of genes involved in the FGP, such as a decrease in the ratio of *Myh6* to *Myh7*, a marker of fetal gene reactivation [23, 24]. Expression of *SERCA2* or *Pln* was not affected by obesity or pregnancy, consistent with preserved systolic function. Curiously, expressions of natriuretic peptides, often used as biomarkers for heart disease [46], were significantly reduced with HF feeding in both nulliparous and postpartum mice. Reduction of cardiac ANP and BNP with obesity has been reported in rodents [47], with leptin described as a key modulator of the anti-hypertrophic effects of ANP in male mice [48]. In the current study, we demonstrate significant LV hypertrophy in obese postpartum female mice where excess weight gain was primarily due to fat accumulation. These data suggest a role for adipocyte-mediated factors in the modulation of cardiac hypertrophy.

Leptin, a hormone secreted by adipocytes in proportion to body mass, may influence cardiac hypertrophy, but conflicting findings are reported [22]. The sequences of leptin and the leptin receptor (and leptin receptor isoforms) are well-conserved among mammals [49]. Tissue expression of the leptin receptor, which includes the heart, and signal transduction is similar between mice and humans [22]. In vitro studies support pro-hypertrophic effects of leptin on cultured rodent and human cardiomyocytes [50–52]. However, whether leptin promotes cardiac hypertrophy in vivo may be dependent on blood pressure, and leptin may have differential effects on blood pressure in humans versus mice. In experimental animals, leptin increases blood pressure via activation of the sympathetic nervous system, but whether this effect is present in humans is inconclusive [53]. Anti-hypertrophic effects of leptin are reported in *ob*/*ob* mice (which are normotensive) [54], and in epidemiologic studies, association of leptin with LV hypertrophy was positive in hypertensive populations [55, 56], and negative in healthy populations [57, 58]. Thus, it does appear that both leptin and blood pressure contribute to cardiac hypertrophy with obesity in mice and humans. Whether leptin contributes indirectly to cardiac hypertrophy via hemodynamic effects in humans is not clear. In the current study, plasma leptin concentrations, markedly increased with HF feeding, were strongly correlated with LV mass in HF-fed but not LF-fed mice. However, plasma leptin concentrations were not augmented in HF-fed postpartum mice with significantly increased LV mass compared with HF-fed nulliparous mice. Importantly, plasma leptin also increases during pregnancy [59]. It is possible that the potential effects of leptin to promote cardiac hypertrophy during pregnancy contributed to enhanced cardiac hypertrophy in HF-fed postpartum mice. Several studies demonstrate the protective effects of adiponectin, an adipocyte-secreted hormone with anti-inflammatory and insulin-sensitizing effects [60], on cardiac hypertrophy [61] and fibrosis [62]. We observed a modest, but significant, increase in plasma adiponectin concentrations in LF-fed but not HF-fed postpartum mice compared with nulliparous controls. Contrary to epidemiologic studies demonstrating a negative association between plasma adiponectin and LV mass [63, 64], we did not observe a correlation among plasma adiponectin and LV mass in any of the groups of mice. As plasma adiponectin concentrations were not different in LF- versus HF-fed mice, results from the current study do not support a role for adiponectin in obesity-mediated cardiac hypertrophy in female mice. Whether changes in adipokines with obesity during pregnancy play a role in pathological cardiac hypertrophy has not been explored.

In the current study, HF-fed postpartum mice with increased LV mass did exhibit increased CO and SV. This is likely a reflection of increased body mass and blood volume with obesity in HF-postpartum mice and was not accompanied by a change in systolic function. A limitation of our study is that we did not assess diastolic function. Although long-term effects of obesity during pregnancy are not known, a recent study reported increased diastolic dysfunction at term in obese versus non-obese pregnant women [19]. LV diastolic dysfunction is more pronounced in women [65]. This may contribute to the increase in risk for developing heart failure with preserved ejection fraction (HFpEF), where female sex and BMI are strong risk factors [66, 67]. Additional studies are needed to determine if cardiac remodeling after pregnancy in obese mice (and humans) is associated with diastolic dysfunction.

Hypertension and obesity independently promote cardiac hypertrophy, and the combined effects are additive in both women and men [68, 69]. A limitation of the current study is that we did not measure blood pressure. Thus, the contribution of blood pressure to increased LV mass in obese postpartum vs nulliparous females is not known. This could be important, as previous studies demonstrate female mice are protected from obesity-hypertension due to sex differences in the RAS [70, 71]. It is not known how obesity during and after pregnancy contributes to the pathology and prevalence of hypertension, or if exacerbated postpartum weight gain overwhelms protective effects in females against the development of hypertension. An additional limitation to this study is that pups were culled after birth (from lean and obese dams), and dams did not nurse pups. This could be important as lactation is demonstrated to have positive effects on postpartum weight loss and metabolism [72].

### Perspectives and significance

Women have traditionally been considered as protected against CVD compared with men, until menopause. Disturbingly, the prevalence of CVD in premenopausal women is increasing in the USA [73]. Given the escalating effect of postpartum weight gain to promote obesity, trends of increasing CVD in premenopausal women could largely be attributed to the increasing number of women entering pregnancy with an overweight or obese BMI. For perspective, data from the National Survey of Family Growth indicate that 77% of women aged 25–34 (who currently do not have children) have expectations to become pregnant [74]; 36.5% of women in this age-range are obese [7]. Findings from the current study demonstrate that women who are obese during pregnancy are a target population for early identification of future risk for CVD. Future studies are needed to identify a reasonable time frame to assess for risk factors or start preventative care. From a public health standpoint, women who are obese during pregnancy are a target population for which public health strategies to improve postpartum nutrition and lifestyle could yield significant reductions in the incidence of CVD.

## Conclusions

In conclusion, these results demonstrate that obesity during and after pregnancy promotes marked LV hypertrophy with moderate dilation of the LV chamber. Both nulliparous and postpartum mice exhibited increased wall thickness and fibrosis in the left ventricles. However, the gene expression profile in obese postpartum mice with LV hypertrophy reflected the active remodeling of the ECM. These results suggest that the combined effects of pregnancy and obesity augment cardiac hypertrophy and promote remodeling postpartum. The clinical significance of this study is that the increasing number of women entering pregnancy as overweight or obese may contribute to the increasing prevalence of CVD in premenopausal women.

## Supplementary information

**Additional file 1: Figure S1.** Experimental design. **Table S1.** Composition of low fat and high fat diets obtained from Research Diets, Diet-Induced Obesity (DIO) series. **Table S2.** Compete list of genes and their NCBI RefSeq mRNA accession numbers included in the custom CodeSet designed by Nanostring for gene analysis using the nCounter. *P* values are for between group differences of normalized mRNA counts using two-way ANOVA with between group factors of pregnancy and diet. **Table S3.** Plasma leptin and adiponectin concentrations of LF- and HF-fed nulliparous and postpartum mice at 20 weeks of diet feeding.

## Data Availability

The datasets used and/or analyzed during the current study are available from the corresponding author on reasonable request.
