# Supplementary material for: Cardiac hypertrophy with obesity is augmented after pregnancy in C57BL/6 mice
Source: Biol Sex Differ. 2019 Dec 16;10:59. doi: 10.1186/s13293-019-0269-z (PMC6916003; doi:10.1186/s13293-019-0269-z)
Supplement: Supplementary file 1 — Additional file 1: Figure S1. Experimental design. Table S1. Composition of low fat and high fat diets obtained from Research Diets, Diet-Induced Obesity (DIO) series. Table S2. Compete list of genes and their NCBI RefSeq mRNA accession numbers included in the custom CodeSet designed by Nanostring for gene analysis using the nCounter. P values are for between group differences of normalized mRNA counts using two-way ANOVA with between group factors of pregnancy and diet. Table S3. Plasma leptin and adiponectin concentrations of LF- and HF-fed nulliparous and postpartum mice at 20 weeks of diet feeding. [file 13293_2019_269_MOESM1_ESM.docx]

Supplementary Figure 1: Experimental design

Female, C57BL/6
age = 8 weeks

n=40

LF diet n=20

HF diet n=20

Non-pregnant n=10

Cross with males n=10

Non-pregnant n=9

Cross with males n=11

**4 weeks diet**

-EchoMRI

-Assign pregnant or non-pregnant

**After delivery (mean 9.2 weeks diet)**

-Pups culled and return females to group housing for study duration

**20 weeks diet**

**-**EchoMRI

-Echocardiography

-Euthanasia/sample collection

Supplementary Table 1: Composition of diets

|  | Low Fat (D12450J) | | High Fat (D12492) | |
| --- | --- | --- | --- | --- |
|  | grams (% grams) | kcal (% kcal) | grams (% grams) | kcal (% kcal) |
| Protein | 203 (19) | 0.76 (20) | 203 (26) | 1.05 (20) |
| Carbohydrate | 631 (67) | 2.67 (70) | 198 (26) | 1.05 (20) |
| Fat | 45 (4) | 0.38 (10) | 270 (35) | 3.14 (60) |
| Total | 1055.05 grams | 100% | 773.85 grams | 100% |
| kcal/gram | 3.82 |  | 5.24 |  |

Supplementary Table 2. Genes included in the NanoString custom CodeSet and between-group *P* values

| Pathway | Gene | Accession number | P_LF_ *_versus_* _HF_ | P_NP_ *_versus_* _PP_ |
| --- | --- | --- | --- | --- |
| Extracellular matrix and fibrosis | *Col1a1* | NM_007742.3 | 0.477 | 0.015 |
|  | *Col3a1* | NM_009930.1 | 0.004 | 0.003 |
|  | *Fn1* | NM_010233.1 | 0.119 | 0.882 |
|  | *Map3k7* | NM_172688.2 | 0.005 | 0.587 |
|  | *Mmp2* | NM_008610.2 | 0.009 | 0.185 |
|  | *Mmp3* | NM_010809.1 | 0.680 | 0.036 |
|  | *Mmp9* | NM_013599.2 | 0.136 | 0.049 |
|  | *Smad2* | NM_010754.4 | 0.001 | 0.332 |
|  | *Smad3* | NM_016769.3 | 0.054 | 0.774 |
|  | *Tgfb1* | NM_011577.1 | 0.249 | 0.313 |
|  | *Tgfb2* | NM_009367.1 | 0.083 | 0.783 |
|  | *Tgfb3* | NM_009368.2 | 0.003 | 0.581 |
|  | *Tgfbr2* | NM_009371.2 | <0.001 | 0.482 |
|  | *Tgfbr3* | NM_011578.3 | <0.001 | 0.418 |
|  | *Timp1* | NM_001044384.1 | 0.951 | 0.738 |
| Fetal gene program | *Acta1* | NM_009606.3 | 0.013 | 0.149 |
|  | *Actb* | NM_007393.3 | 0.140 | 0.095 |
|  | *Atp2a2* (SERCA2) | NM_001110140.3 | 0.219 | 0.251 |
|  | *Myh6* | NM_010856.4 | 0.01 | 0.111 |
|  | *Myh7* | NM_080728.2 | 0.597 | 0.031 |
|  | *Pln* | NM_023129.5 | 0.27 | 0.386 |
| Hypertrophy | *Nppa* | NM_008725.2 | <0.001 | 0.03 |
|  | *Nppb* | NM_008726.5 | <0.001 | 0.605 |
|  | *Nppc* | NM_010933.5 | 0.135 | 0.159 |
|  | *Npr1* | NM_008727.5 | 0.12 | 0.694 |
| Angiogenesis | *Angpt1* | NM_009640.3 | 0.002 | 0.622 |
|  | *Angpt2* | NM_007426.4 | 0.985 | 0.205 |
|  | *Ppargc1a* | NM_008904.2 | 0.879 | 0.073 |
|  | *Vegfa* | NM_001025250.3 | 0.088 | 0.13 |
| Renin-angiotensin system | *Ace* | NM_009598.1 | 0.047 | 0.86 |
|  | *Ace2* | NM_001130513.1 | 0.089 | 0.836 |
|  | *Agt* | NM_007428.3 | 0.053 | 0.634 |
|  | *Agtr1a* | NM_177322.3 | 0.297 | 0.811 |
|  | *Agtr1b* | NM_175086.3 | 0.848 | 0.891 |
|  | *Atp6ap2* | NM_027439.4 | 0.982 | 0.929 |
|  | *Mas1* | NM_008552.4 | 0.157 | 0.05 |
|  | *Ren1* | NM_031192.3 | 0.401 | 0.157 |
| Estrogen receptors | *Esr1* | NM_007956.4 | 0.1 | 0.575 |
|  | *Esr2* | NM_010157.3 | 0.443 | 0.424 |
| Housekeeping | *Eef1e1* | NM_025380.2 | 0.744 | 0.171 |
|  | *Gapdh* | NM_008084.1 | 0.484 | 0.24 |
|  | *Rpl4* | NM_024212.4 | 0.477 | 0.333 |
|  | *Ywhaz* | NM_001253806.1 | 0.145 | 0.219 |

Supplementary Table 3: Plasma leptin and adiponectin concentrations of LF- and HF-fed nulliparous and postpartum mice at 20 weeks of diet feeding.

|  | Nulliparous | | Postpartum | |
| --- | --- | --- | --- | --- |
|  | LF | HF | LF | HF |
| Leptin (ng/ml) | 4.38 + 1.02 | 67.43 + 14.39^*^ | 6.39 + 1.24 | 76.38 + 10.36^*^ |
| Adiponectin (ug/ml) | 12.71 + 0.38 | 13.18+ 0.44 | 14.24 + 0.38^#^ | 14.36 + 0.60 |

*, P<0.001 effect of HF diet analyzed by 2-way ANOVA
#, P<0.05 effect of postpartum analyzed by 2-way ANOVA
